# Supplementary material for: Reinfection with SARS-CoV-2 and Waning Humoral Immunity: A Case Report
Source: Vaccines (Basel). 2022 Dec 20;11(1):5. doi: 10.3390/vaccines11010005 (PMC9861578; doi:10.3390/vaccines11010005)
Supplement: Supplementary file 1 [file vaccines-11-00005-s001.zip › vaccines-2071008-supplementary-xml (1).pdf]

Reinfection with SARS-CoV-2 and Waning Humoral Immunity: A Case Report.

Supplement:

Methods

Figure S1: Flow Diagram of Shedding and “Re-positives”

Figure S2: Chest Radiography at Primary Infection and Reinfection

Table S1: Complete PCR testing history for SARS-CoV-2

Table S2: Intra-host Single Nucleotide Variants

Figure S3: Titration curves for Anti-SARS-CoV-2 Serologies

Figure S4: Anti-SARS-CoV-2 IgG subclass distribution

Figure S5: Pseudovirus Neutralization IC50 curves

Table S3: Sequence Acknowledgement Table

Supplemental Methods:

Labcorp, UW Virology, ISB, rt-PCR Testing:

Quantitative real-time polymerase chain reaction (rt-PCR) to generate the population rt-PCR data (**Figure 1**) and for the case patient clinical and research diagnostic testing (**Table 1** and **Table S1**) was performed in three laboratories: Labcorp, University of Washington (UW) Virology and Institute for Systems Biology (ISB). Assays for rt-PCR are comparable across platforms [33,34].

Population rt-PCR data (**Figure 1**): Ct were obtained from the Cepheid Infinity at Labcorp Microbiology at Swedish Health Services on all persons tested since the start of the pandemic. Figures and descriptive statistics were generated in Stata v13.1 (StataCorp LP, College Station, TX). The Ct of each target was averaged and displayed in a spaghetti plot, except where only one target was detectable and then the single positive target was displayed. Ct ranged from 14.9 – 44.0 and negative (undetectable) results on both targets were assigned a value of CT = 50 for the purpose of data visualization.

Clinical care diagnostic testing: In March, the CTs were 22.8 (E gene) and 26.5 (RdRp gene) at 8 days after symptoms onset (**Table 1**) and were performed on a lab-developed test at UW Virology in Seattle, WA based on the WHO primers [35]. In July, on the first nasopharyngeal swab, CTs were 43.3 (E gene) and 39.6 (N2 gene) on day 14 after symptoms onset from reinfection (**Table 1**). This test was performed at LabCorp Microbiology, on-site at Swedish Health Services in Seattle, WA with the Xpert Xpress SARS-CoV-2 test on the GeneXpert Infinity System (Cepheid, Sunnyvale, CA), as per the manufacturer guideline.

Research diagnostic testing: Next, to confirm the diagnosis of SARS-CoV-2 in July, a separate swab was collected for research purposes by a nurse on a mid-turbinate swab on day 19 after symptoms onset from reinfection (**Table S1**). Ct of 38.5 was obtained, as performed on a lab-developed test at ISB in Seattle, WA based on the CDC primers for N1 gene [36]. Whole viral genome sequencing in March was performed on the sample sent to UW Virology and in July was performed on the sample sent to ISB.

To assure samples are not contaminated at ISB, standard practices are applied including physical separation of pre-PCR and post-PCR workspace. All reactions are set up in the clean PCR hood with separate equipment and consumables, UV light, air control, use of aerosol resistant pipettes and dedicated PCR mastermix. There is unidirectional work flow from pre-PCR to post-PCR with personal protective equipment (PPE) changed and hand hygiene performed. In no circumstance is a post-PCR amplicon ever brought into the pre-PCR workspace.

UW Virology rapid metagenomic NGS:

In March, rapid metagenomic next-generation sequencing (NGS) was performed [18,37]. Briefly, nasopharyngeal swab samples were filtered, RNA was extracted, then treated with DNAse. Libraries were generated from cDNA and 20 cycles of PCR, then quality controlled prior to sequencing run on MiSeq desktop (Illumina, San Diego, CA) using 180 cycles single-read reagent kit. Trimmed and quality filtered reads were assembled de novo, contigs aligned to the reference sequence (NC_045512), and a consensus sequence was generated. The sequence was deposited in Genbank (MT252824) and GISAID (EPI_ISL_416456). For Table S2, to calculate the percent of reads for the SNV, raw reads were mapped to the reference sequence (NC_045512).

Institute for System Biology multiplex PCR amplicon NGS:

In July, highly multiplexed PCR amplicon NGS was performed on the D16 nasopharyngeal swab, using the ARTIC V3 amplicon primer scheme [19], and modified from the ARTIC protocol [38], to approach the low copy number sample, as follows. RNA was extracted from nasopharyngeal swab samples using the miRNeasy kit (Qiagen, Germantown, MD) and cDNA was synthesized with the LunaScript RT SuperMix kit (NEB, Ipswich, MA). The set of 98 SARS-CoV-2-specific primer pairs were synthesized [19] and pooled for multiplex PCR using the following PCR conditions: 98°C for 30 seconds, followed by 40 cycles of 95°C for 15 seconds, 65°C for 5 minutes. The primer set generated overlapping PCR fragments covering the entire viral genome, the average amplicon length being 400 bp with an approximate 100 bp overlap. After cleaning up the PCR products with AMPure XP beads (Beckman Coulter, Indianapolis, IN), Fragmentase (NEB, Ipswich, MA) was added to break down the PCR products to about 200 bp for library construction. The sequencing libraries were prepared with NEBNext Ultra II kit for Illumina (NEB, Ipswich, MA) following manufacturer’s protocol and each library was assigned with a unique 6-base index. Libraries ranging in size from 300 to 500 bp were selected with Pippin HT (Sage Science, Beverly, MA) and the size was confirmed with the Bioanalyzer (Agilent, Santa Clara, CA). Libraries were quantified with the KAPA Library Quantification Kit for Illumina Platforms (Kapa Biosystems, Wilmington, MA) and pooled at a final concentration of 5nM. Pooled libraries were run on NextSeq (Illumina, San Diego, CA) using 300 cycles paired end reagent kit. The raw sequence data was demultiplexed using qcat (https://github.com/nanoporetech/qcat) and assembled against reference genome (NC_045512) with Sequencher (Gene Codes, Ann Arbor, MI), allowing for no more than 2 mismatches within a 40 base pair window. Ambiguous bases were amplified via PCR and sent for Sanger sequencing. The mutation at C18877T is confirmed via Sanger sequencing (Primers: Forward CGCACCTGTTGTCTATGTGA, Reverse: GAACCTTTCTACAAGCCGCAT, Sequencing: GGTTTTACAGGTAACCTACA), but amplification was unsuccessful at other positions. To avoid contamination, the same practices were utilized for library preparation as described above (in PCR) including physical separation of pre-PCR and post-PCR spaces, PPE and techniques.

Variant Calling and Phylogenetic Analysis:

Single nucleotide variants (SNV) are given for any positions with variants compared to Wuhan-Hu-1 reference with any % mapped reads > 5% (**Table S2**). For the purposes of the phylogeny, SNVs were confidently called only if the variant existed in >90% of mapped reads. Consensus viral sequences from InCoV139-March and InCoV139-July were analyzed using Nextstrain software (build 8f28f11) [20]. NextClade was used to determine differences from the reference sequence and to ascertain clade [39], using Nextrain's taxonomy of clades: 19A, 19B, 20A, 20B, and 20C. We included representative Washington sequences present in GISAID [40] together with non-Washington sequences illustrative of major root and intermediate clade nodes for context. For comparison, the pairs of sequences from the two other reinfection cases from Hong Kong and Nevada were also included [10,11]. The sequence acknowledgement table for included samples is given (**Table S3**). Alignment and tree construction proceeded with the default parameters for the Nextstrain pipeline (build 8f28f11); trees were visualized with auspice (auspice.us) and Tree of Life [41]. In a sensitivity analyses, ambiguous variants in the July sequence were called with lower SNV thresholds. These analyses could change the clade determination from 20A to 20C, but do not change the phylogenetic conclusion that InCoV139-July (clade 20A or 20C) is distinct from InCoV139-March (clade 19B).

ISB, Plasma and PBMC isolation:

Plasma and PBMC isolation were conducted with standard protocols from Bloodworks Northwest (Seattle, WA). Patient blood were collected in BD Vacutainer (EDTA) tubes (Becton, Dickinson and Company, Franklin Lakes, NJ). Plasma fractions were collected after centrifuged at 800 x g at 4°C for 10 min, aliquoted and stored until use at -80°C. The rest of the blood were diluted with PBS (pH7.2) to 2X of original volume and layered over 15 ml Ficoll (GE Healthcare, Waukesha, WI) in SepMate-50 tubes (Vancouver, BC). After centrifuged at 800 x g for 15 min at room temperature, the PBMC layer (did not include granulocytes (such as neutrophils)) was poured into a 50 ml conical tube. The cells were washed twice with autoMACS Rinsing Solution (Miltenyi Biotec, Auburn, CA) and centrifuge at 250 x g for 10 min, at RT. PBMC pellets were gently resuspended in 5 ml Rinsing Solution and a 5 μl aliquot was diluted 1:10 v/v for cell counting. Cells in 18 μl of diluted samples were first mixed with 2 μl of Acridine Orange / Propidium Iodide Stain (Logos Biosystems, Annandale, VA), 10 μl was then loaded to a PhotonSlide (Logos Biosystems) and counted in a LUNA FL Dual Fluorescence cell counter (Logos Bioystems). Cryopreservation freeze media CryoStor CS-10 (Biolife Solutions, Bothell, WA) was slowly added to make a concentration of 2.5 million PBMC/ml. Cells were aliquoted in Cryotube vials (ThermoFisher, Waltham, MA) and frozen in CoolCell LX Cell Freezing Container (Corning, Corning, NY) at -80°C for at least 2 hours before stored until use in LN.

Stanford anti-SARS-CoV-2 ELISA serology and ACE2-RBD blocking antibody assay:

ELISAs with SARS-CoV-2 antigens were performed using protocols as previously described [13]. Briefly, 96-well plates that were coated with SARS-CoV-2 RBD, full length spike, or nucleocapsid (N) antigens were incubated with 2-fold serial dilutions of patient plasma samples from 1:50 to 1:3200 alongside a negative and positive plasma control for each plate. Bound antibodies were detected with peroxidase-conjugated anti-human IgG, IgM, or IgA antibodies. IgG subclass assays were completed with 1:100 diluted plasma samples using peroxidase-conjugated anti-human IgG1, IgG2, IgG3, or IgG4 antibodies. A chromogenic reaction was initiated with TMB and quenched with sulfuric acid. The optical density (OD) at 450 nm was read with a microplate reader and adjusted for background OD from blank wells. The cutoff values for positivity were defined as 3 SDs above the mean ODs from pre-pandemic healthy blood donors. Competition ELISAs to detect antibodies blocking binding of ACE2 to RBD were performed. Briefly, 96-well plates coated with SARS-CoV-2 RBD were incubated with patient plasma samples at a dilution of 1:10, alongside a negative and positive plasma control for each plate and with soluble human ACE2 fused to a mouse Fc (ACE2-mFc). The Fc region of ACE2-mFc was detected with peroxidase-conjugated goat anti-mouse IgG. The chromogenic reaction and reading of OD were performed in the same manner as for the Ig isotype and IgG subclass assays. The percentage of ACE2-RBD blocking by anti-RBD plasma antibodies was calculated as the relative decrease in ACE2 binding compared to a negative quality control plasma included in each experiment. Data were analyzed and plotted in GraphPad Prism version 8.0.

Monogram Biosciences, pseudovirus neutralization (PhenoSense® CoV Neutralizing Antibody Assay):

A cell-culture based assay to test neutralizing activity of patient plasma to pseudovirus infection was performed using protocols as previously described [21]. Briefly, a lentivirus-based SARS-CoV-2 pseudovirus particle was generated expressing spike proteins on the surface from the Wuhan-Hu-1 reference (Genbank: NC_045512) expressing the early D614. A separate pseudovirus particle expressing the transitioned D614G mutation was engineered from site directed mutagenesis on the original Wuhan-Hu-1 strain. Plasma samples were heat inactivated at 56°C for one hour and diluted 1:40 in cell culture medium. Neutralizing antibody (nAb) titers were determined by creating 9 serial three-fold dilutions of test samples which were mixed with ~105 relative light units (RLU) of SARS-CoV2 pseudotyped virus and incubated at 37°C for one hour. Separately, irrelevant virus was used as a specificity control and mixed with test samples. Following the one-hour incubation HEK 293 ACE2-transfected cells were added to the well. The plates were incubated for 60-80 hours at 37°C and then assayed for luciferase expression. Neutralization titers are reported as the reciprocal of the serum dilution conferring 50% inhibition (ID50) of pseudovirus infection, adjusting for background florescence. SARS-CoV-2 nAb assay positive and negative control plasma were included on each 96-well assay plate (1 each) along with 6 patient specimens.

Stanford, IGH sequencing and repertoire analysis:

Immunoglobulin heavy chain (IGH) libraries were prepared for high throughput sequencing from cDNA generated from total RNA isolated from PBMCs. Reverse transcription was primed with random hexamer primers, and cDNA was used as template for PCR amplification using barcoded IGHV FR1 primers and isotype specific primers located in the first exon of the constant region for each isotype category (IgM, IgD, IgE, IgA, IgG), with each isotype amplified in a separate reaction, as previously reported [42]. Libraries were pooled, gel purified and quantitated prior to sequencing on MiSeq (Illumina), using 600 cycle kits for paired end 300 cycle sequencing. Read pairs were joined and then aligned to IGH germline gene segments using IgBLAST [42,43]. Exact matching to non-primer encoded constant region gene sequence was required for isotype identification. Clonal identities within each subject were inferred using single linkage clustering and the following definition: same IGHV and IGHJ usage (disregarding allele call), equal CDR-H3 length, and minimum 90% CDR-H3 nucleotide identity. Somatic mutation frequency for clones was quantitated with reference to the best matching IGHV germline gene. Data were plotted in R. Single-cell immunoglobulin libraries were prepared using the 10x Single Cell Immune Profiling Solution Kit (v1.1 Chemistry), according to the manufacturer's instructions. Single-cell Ig libraries were sequenced on an Illumina MiSeq to a minimum sequencing depth of 5,000 reads/cell. Consensus Ig annotation was performed using cellranger vdj (10x Genomics, version 4.0.0 with GRCh38/Ensembl reference data). Further details for repertoire analysis are available in Nielsen et al. [22].

ISB, Single cell multi-omics assays:

Chromium Single Cell Kits (10x Genomics) were utilized to analyze the transcriptomic, surface protein levels and TCR and BCR sequences simultaneously from the same cell. Experiments were performed according to manufacturer's instructions.

Briefly, cryopreserved PBMCs were thawed and 1X red blood cell lysis solution (BioLegend) was used to lyse any remaining red blood cells in the PBMC samples. Cells were stained with a panel of TotalSeq-C human antibodies that includes hashtag multiplexing antibodies (BioLegend) according to manufacturer's protocol. Stained cells were then loaded onto a Chromium Next GEM chip G. Cells were lysed for reverse transcription and complementary DNA (cDNA) amplification in the Chromium Controller (10X Genomics). The polyadenylated transcripts were reverse-transcribed inside each gel bead-in-emulsion afterwards. Full-length cDNA along with cell barcode identifiers were PCR-amplified and sequencing libraries were prepared and normalized. The constructed library was sequenced on Novaseq platform (Illumina).

ISB, Single cell RNA-seq data processing:

Droplet-based sequencing data were aligned and quantified using the Cell Ranger Single-Cell Software Suite (version 3.0.0, 10x Genomics) against the GRCh38 human reference genome. Cells from each demultiplexed sample were first filtered for cells that expressed

a minimum of 200 genes, then they were filtered based on three metrics: 1) the total number of unique molecular identifiers (UMI) counts per cell (library size) must be less than 10000; 2) the number of detected genes per cell must be less than 2500; and 3) the proportion of mitochondrial gene counts (UMIs from mitochondrial genes / total UMIs) must be less than 10%. Doublets were either simultaneously identified in sample demultiplexing or identified using scrublet [44] and were removed prior to aforementioned filtering. After QC metric filtering, a total of 226,288 cells were retained for downstream analysis. Scanpy [45] was used to normalize cells via CPM normalization (UMI total count of each cell was set to 10^6) and log1p transformation (natural log of CPM plus one).

<u>ISB, Single cell RNA-seq cell type identification dimensional reduction and clustering</u>:

PCA was performed on the normalized, ln(CPM+1), gene expression matrix of all cells passing the previously mentioned QC metrics, “arpack” was set as the SVD solver. A neighborhood graph was built with n-neighbors set to 15 and all 50 calculated PCs as inputs. This neighborhood graph was utilized to calculate cell type clusters via the Leiden algorithm [46]. Clusters identified in this first round of clustering were annotated based on the expression of canonical marker genes. Clusters that were not uniform in their expression of well-known marker genes were extracted and a second round of dimension reduction and clustering was performed on these subsets. Clusters that simultaneously expressed canonical markers from two or more major cell types were identified as potential doublets or low-quality cells and were removed from downstream analysis. 2,106 low-quality cells were removed resulting in 224,182 total cells for further analyses.

Identified T cells from the first and second round of clustering were extracted for $CD4^+$ and $CD8^+$ T cell identification. A $CD4^+$ T cell score was obtained by taking the sum of the scaled values (scale the ln(CPM + 1) values to be from 0 to 1) of the *CD4* transcript and CD4 surface protein. A $CD8^+$ T cell score was obtained by taking the sum of the scaled values of the *CD8A* and *CD8B* transcripts, and the CD8 surface protein. Both scores were subsequently scaled to be from 0 to 1 and utilized as inputs for a scatterplot for manual gating of $CD4^+$ T cells and $CD8^+$ T cells. Other T cells were then defined as T cells that did not confidently categorize as $CD4^+$ or $CD8^+$.

Gene expression is shown in relative abundance on the heatmap to phenotype CD8+ T cells and CD4+ T cells. Markers are chosen to describe CD8+ T cell subsets including: naive, memory, effector, proliferative, and exhausted. Markers are chosen to describe CD4+ T cells subsets including naive, T helper cell 1 (Th1), cytotoxic, regulatory T cells (Treg), follicular helper T cells (Tfh), proliferative, and exhausted.

World Health Organization (WHO) ordinal scale score to characterize disease severity at the time of sample collection was applied as follows at the time of sample collection: WHO 1-2: ambulatory; WHO 3: hospitalized, not requiring supplemental oxygen; WHO 4: hospitalized, requiring supplemental oxygen by nasal cannula; WHO 5: hospitalized, requiring supplemental oxygen by high flow nasal cannula; WHO 6: hospitalized, requiring

invasive mechanical ventilation (IMV); WHO 7: hospitalized, requiring IMV and organ failure (ECMO, vasopressors or dialysis).

ISB Plasma proteomics:

Plasma concentrations of proteins were measured using the ProSeek Cardiovascular II, Inflammation, Metabolism, Immune Response, and Organ Damage panels (Olink Biosciences, Uppsala, Sweden). Healthy control plasma samples were processed at Olink facilities in Boston, MA. Plasma samples from COVID-19 participants were assayed at the Institute for Systems Biology. Proteins from patient plasma were measured using proximity extension assay (PEA) (Olink Proteomics, Uppsala, Sweden) which allows for the simultaneous analysis of 92 protein biomarkers on each panel. Five panels including Inflammation, Cardiovascular II, Organ Damage, Immune Response and Metabolism were run using patient plasma samples as well as 8 replicates of a pooled healthy control. One microliter of plasma was incubated overnight and allowed to bind with oligonucleotide-labeled antibody pairs to form specific DNA duplexes. This template was then extended and pre-amplified, and the individual protein markers were measured using high-throughput microfluidic real-time PCR. The resulting Ct values were normalized against an extension control, an inter-plate control, and adjusted with a correction factor according to the manufacturer's instructions to calculate a normalized protein expression value (NPX) in log2 scale.

Samples were processed in batches with pooled quality control samples included in each batch; potential batch effects were subsequently adjusted using the pooled control samples, as previously described [47]. Plasma proteins are chosen as representative markers of inflammation (IL-6), stimulatory (IL-8), regulatory (IL-10, IL-4), and chemoattractive (CCL2) cytokines. We also included VEGF level for comparison given the key role of this marker in hypoxia.

Supplemental Figure Legends

Figure S1: Flow Diagram for Repositive Analysis. All PCR samples for hospitalized or Emergency Department patients were analyzed. Unique patients were selected from all samples in subsequent steps who had 1) any single positive test, 2) two or more positive tests (n=176 for shedding duration analysis, **Figure 1A**), and 3) at least three tests in a "re-positive" pattern of positive – negative – positive (n=43 for re-positive analysis, **Figure 1B**).

Figure S2: Chest Radiography. A: March Admission, Hospital Day #5 showing hyperinflation of emphysema and bilateral lower lobe infiltrates. B: July Admission, Hospital Day #1: showing baseline emphysema without acute cardiopulmonary disease.

Figure S3: Titration curves for IgG, IgM and IgA specific for RBD, Spike and N antigens. Serial twofold plasma dilutions were assayed to determine anti-spike (A), anti-RBD (B), and anti-N (C) IgM, IgG, and IgA antibody titers. Plasma pools from SARS-CoV-2 pre-pandemic healthy blood donors and from COVID-19 patients were used as negative and positive quality control (QC), respectively. Assays were performed in duplicate and mean OD values are shown.

Figure S4: Anti-SARS-CoV-2 IgG subclass distribution. Plasma samples collected at D14, D18, D21, and D42 after reinfection COVID-19 diagnosis were analyzed at a 1:100 dilution for their anti-spike (A), anti-RBD (B), and anti-N (C) IgG subclass profile. A SARS-CoV-2 pre-pandemic plasma pool from healthy blood donors was included as negative quality control (QC). Assays were performed in duplicate and mean OD values are shown.

Figure S5: Pseudovirus Neutralization IC50 curves. Columns represent the dilution curves for patient sample at day 14 after reinfection and day 42 after reinfection. Rows represent pseudovirus assays expressing the wildtype SARS-CoV-2 D614 (of the 19A clade), newer SARS-CoV-2 G614 (of the 20A clade), and SARS-CoV-1 (2003). Serial three-fold plasma dilutions starting at 1:40 were pre-incubated separately with pseudovirus particles prior to adding ACE2-expressing HEK 293, and the cell cultures were read at 72 hours. The plasma dilution needed to inhibit 50% of infectivity (IC50%) was read from the sigmoidal inhibition curves.

Figure S1. Flow Diagram of Shedding and "Re-positives"

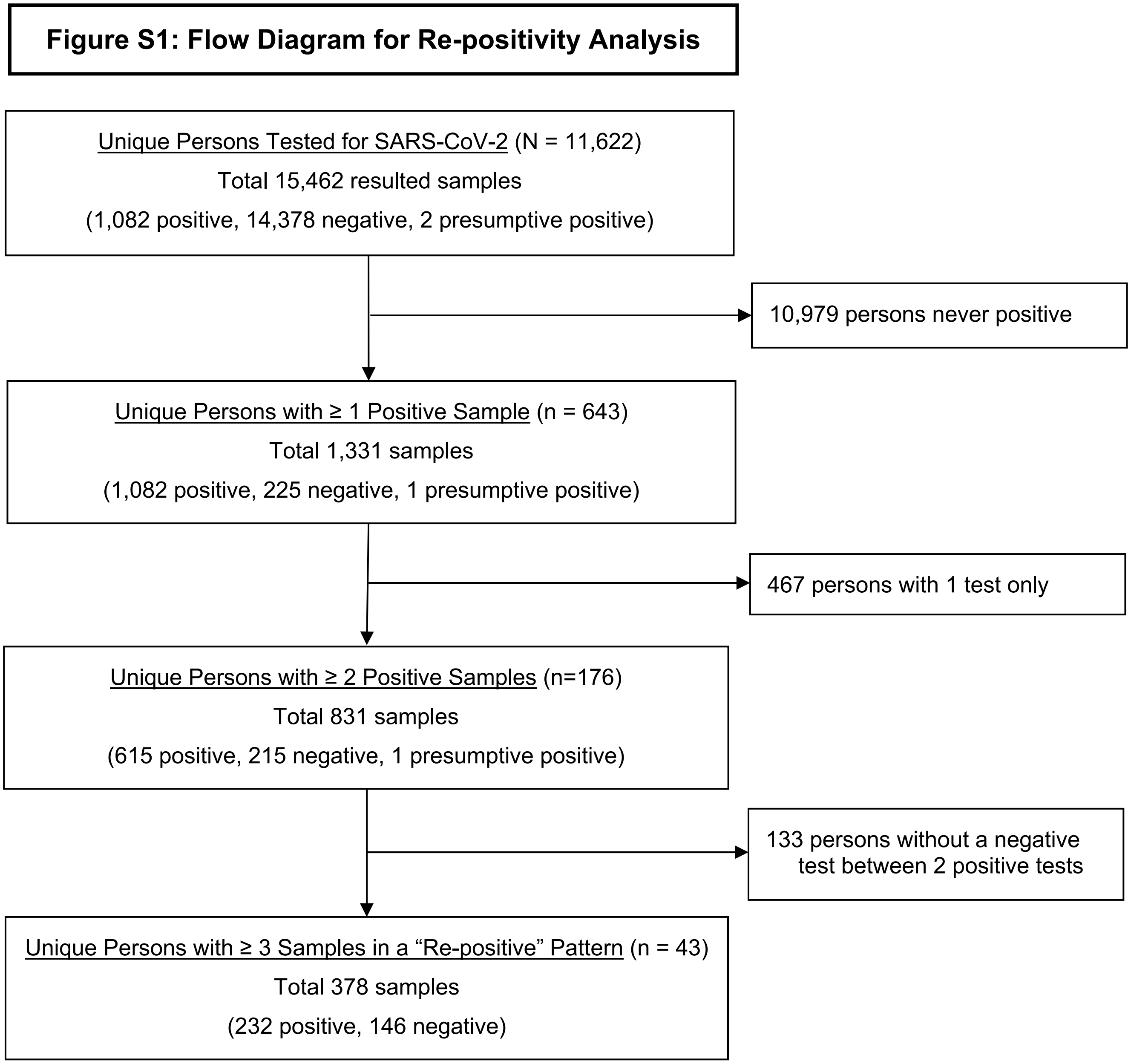

Figure S2. Chest Radiography. A: March Admission, HD#5 showing hyperinflation of emphysema and bilateral lower lobe infiltrates. B: July Admission, HD#1: showing baseline emphysema without acute cardiopulmonary disease.

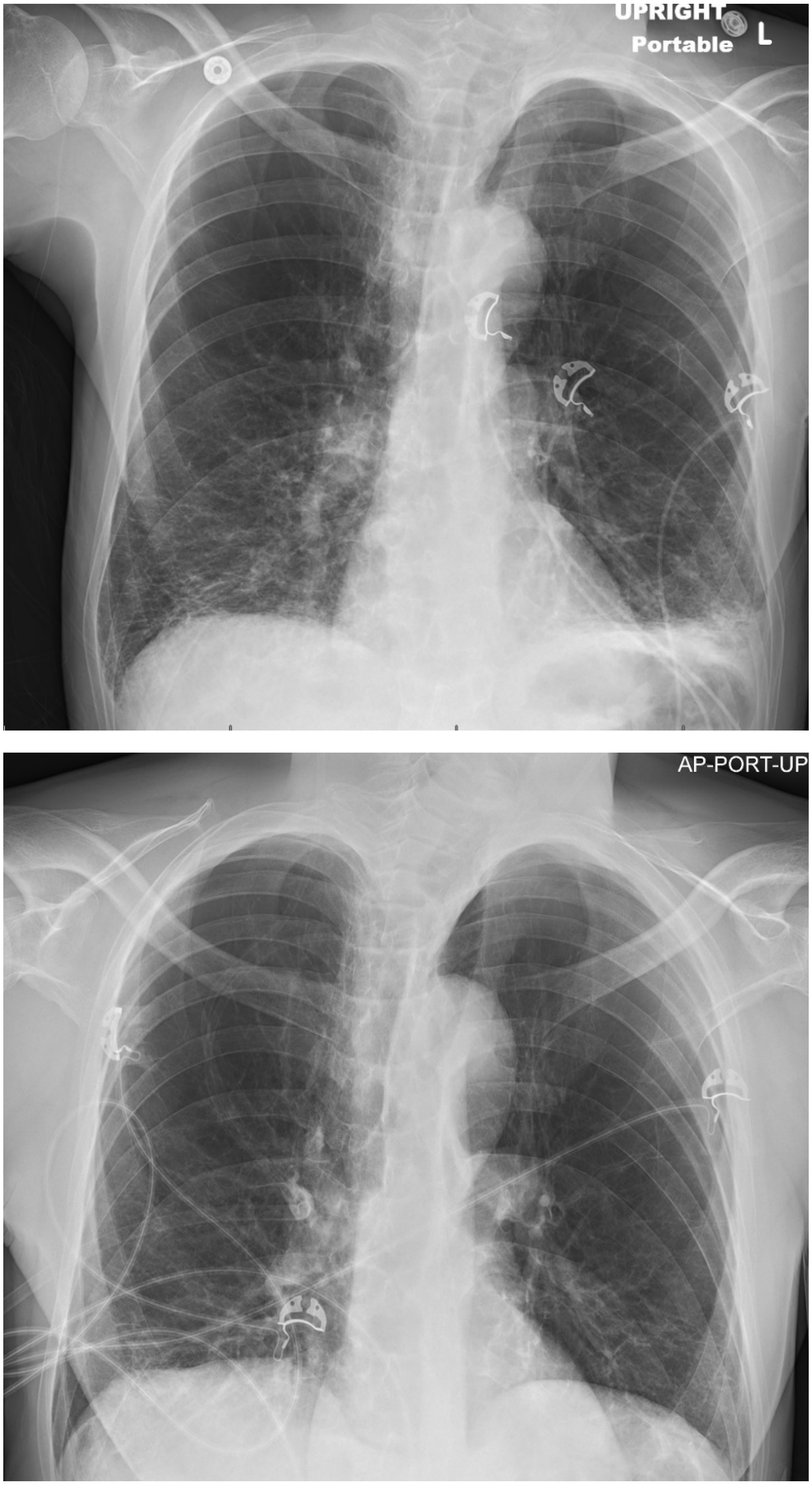

Table S1. Complete PCR testing history for SARS-CoV-2.

Timeframe*		Characteristics of rt-PCR Test		Results		Sample Types**	
Primary Infection	Reinfection	Testing Platform (manufacturer)	Targets	Qual	CT	Purpose	Swab
8	-126	LDT (UW)	E, RdRp	+	22.8, 26.5	Clinical	NP
20	-114	LDT (Labcorp)	N1, N2	+	N/A	Clinical	NP
23	-111	Cobas (Roche)	Orf1ab, E	+	N/A	Clinical	NP
28	-106	Panther (Hologic)	Orf1ab/2ab	+	35.3	Clinical	NP
34	-100	Infinity (Cepheid)	E, N2	+	36.3, 39.5	Clinical	NP
39	-95	Infinity (Cepheid)	E, N2	+	35, 37.4	Clinical	NP
43	-91	Infinity (Cepheid)	E, N2	-	ND, ND	Clinical	NP
45	-89	Infinity (Cepheid)	E, N2	-	ND, ND	Clinical	NP
148	14	Infinity (Cepheid)	E, N2	+	43.3, 39.6	Clinical	NP
149	15	RUO (ISB)	N1	-	ND	Research	MT
150	16	Infinity (Cepheid)	E, N2	-	ND, ND	Clinical	NP
151	17	Infinity (Cepheid)	E, N2	-	ND, ND	Clinical	NP
153	19	RUO (ISB)	N1	+	38.5	Research	MT
175	41	Infinity (Cepheid)	E, N2	-	ND, ND	Clinical	NP
176	42	RUO (ISB)	N1	-	ND	Research	MT

Abbreviations: rt-PCR = real time polymerase chain reaction; Qual = qualitative; CT = Cycle Threshold; N/A = not available; ND = not detected; LDT = lab developed test; RUO = research use only; ISB = Institute for Systems Biology; UW = University of Washington; NP = nasopharyngeal; MT = mid-turbinate.

* Time frame is given from the date of symptoms onset for each infection. Primary infection was diagnosed in early March and reinfection was diagnosed in late July.

** All samples were nurse collected. The research protocol allows for patient self-collection, but the case patient requested nurse collection. Nasopharyngeal (NP) is the protocol for clinical care sample collection and mid-turbinate (MT) is the protocol for research sample collection.

Table S2. Intra-host Single Nucleotide Variants.

Mutation	Date	SNV	Clade	Protein	Substitution	% reads for SNV
1	March	C8782T	19B	Nsp4	-	1.00
2	March	C17747T	19B	Helicase	P1427L	1.00
3	March	A17858G	19B	Helicase	Y1464C	1.00
4	March	C18060T	19B	Exonuclease	-	1.00
5	March	T28144C	19B	orf8	L84S	1.00
1	July	C241T	20A	5'UTR	-	1.00
2	July	C1059Y	20C	Nsp2	T85I	0.19
3	July	C3037T	20A	Nsp3	-	1.00
4	July	C6539Y	unique	Nsp3	H2092Y	0.64
5	July	G11540K	unique	Nsp6	V3579F	0.38
6	July	A12557M	WA outbreak	Nsp8	I4098L	0.27
7	July	C14408T	20A	RdRp	P314L	1.00
8	July	C18877T*	20A	Exonuclease	-	0.57
9	July	A23403G	20A	S protein	D614G	1.00
10	July	G25483R	unique	orf3a	A31T	0.60
11	July	G25563T	20C	orf3a	Q57H	0.94
12	July	C27167Y	unique	M protein	-	0.52

* Ambiguity code not given because SNV confirmed with Sanger sequencing.

Canonical mutations are colored in the above table when passing the variant calling method, as follows from this key:

Color:	Clade:	Ancestor:	Canonical Mutations:	Node Viruses:
	19A	Root	Reference	Wuhan-Hu-1
	19B	19A	C8782T, and T28144C	WA1 shares C18060T
	20A	19A	C3037T, C14408T, A23403G	Germany/BavPat3-ChVir1020
	20B	20A	G28881A, G28882A, G28883C	
	20C	20A	C1059T, and G25563T	

Figure S3. Titration curves for IgG, IgM and IgA specific for RBD, Spike and N antigens.

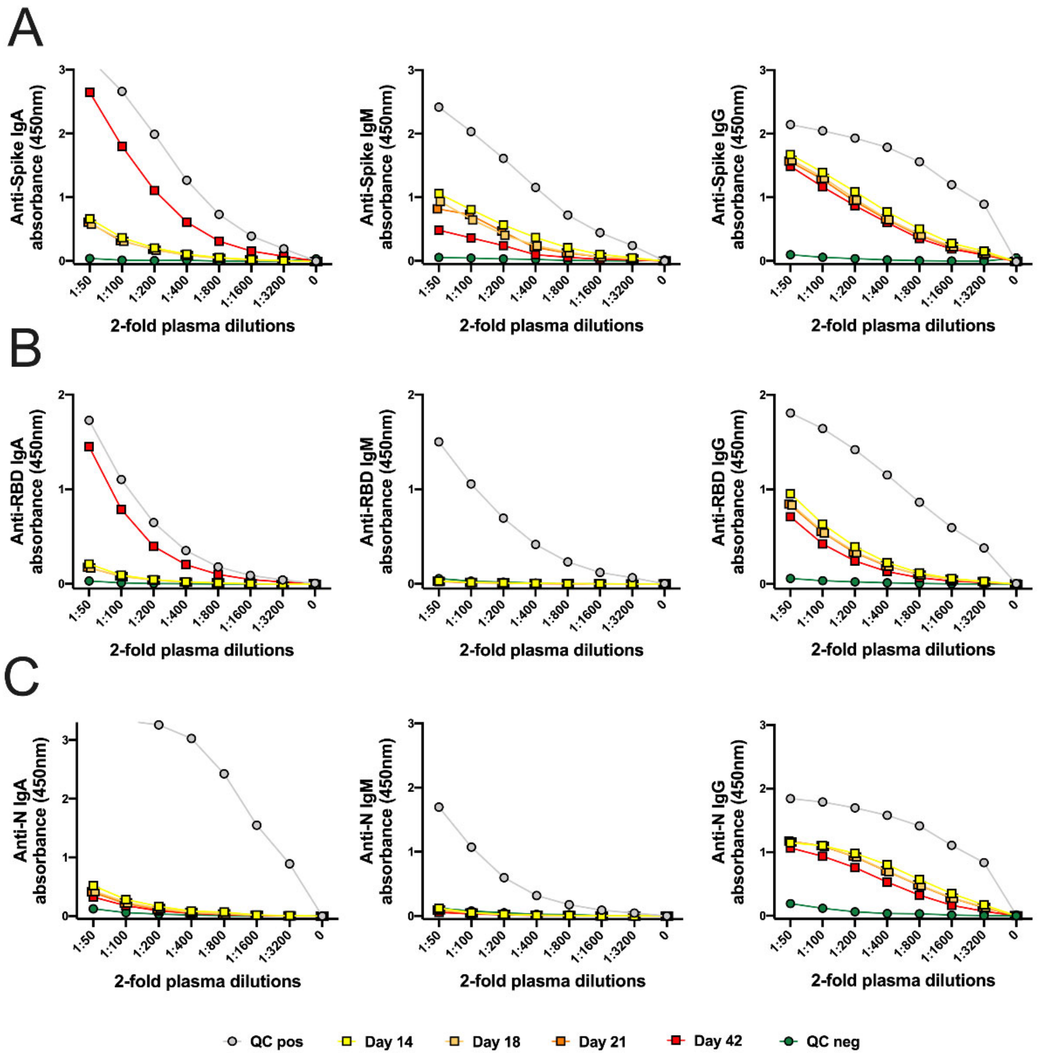

Figure S4. Anti-SARS-CoV-2 IgG subclass distribution.

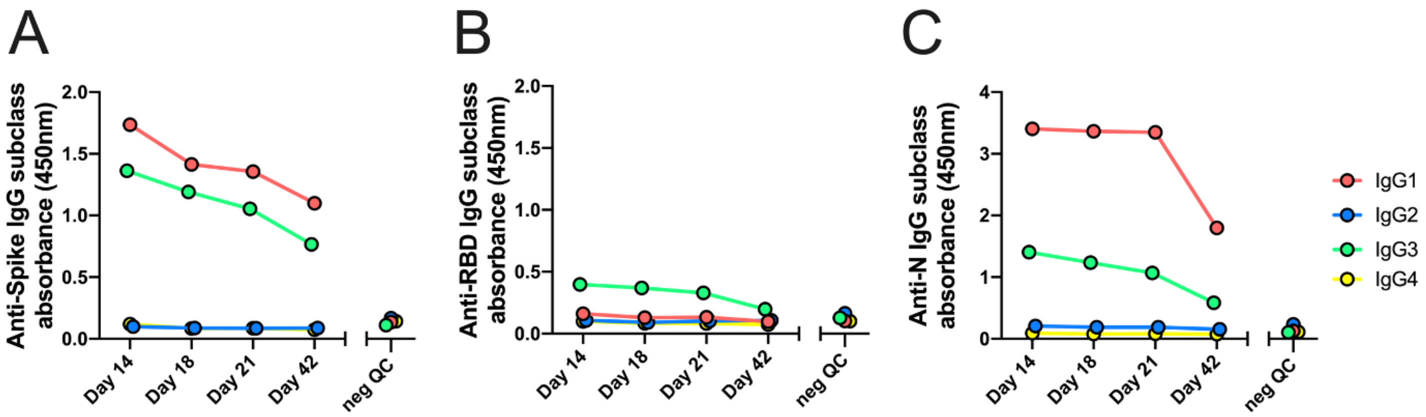

Figure S5. Pseudovirus Neutralization IC50 Curves.

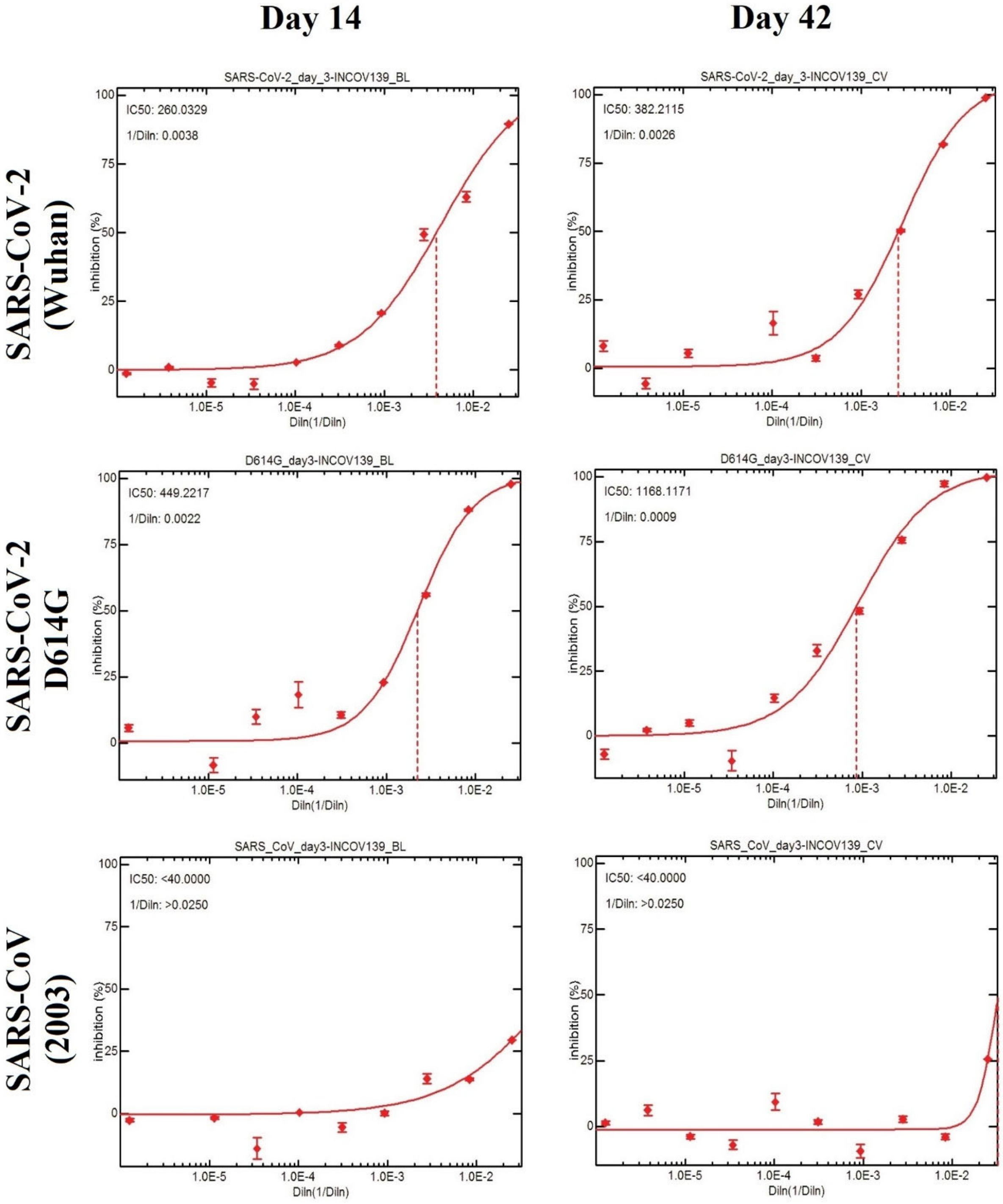

Table S3. Sequence Acknowledgement Table.

GenBank / Name	GISAID Accession	Collection Date	Originating / Submitting Lab	Authors:
Wuhan/Hu-1/2019	EPI_ISL_402125	12/26/2019	China CDC / same	Zhang et al
USA/WA1/2020	EPI_ISL_404895	01/19/2020	PRMCE / US CDC	Queen et al
Australia/NSW2153/2020	EPI_ISL_509505	01/25/2020	SAViD / same	Rawlinson et al
Germany/BavPat3-ChVir1020/2020	EPI_ISL_450200	01/28/2020	Dept Virology / same	Boehmer et al
France/HF1463/2020	EPI_ISL_429968	02/21/2020	CHCLB / NRCVRI	Albert et al
USA/WA-S82/2020	EPI_ISL_417135	02/22/2020	WA DOH / SFS	Chu et al
USA/WA3-UW1/2020	EPI_ISL_413025	02/27/2020	HMC / UW Virology	Roychoudhury et al
USA/WA-S47/2020	EPI_ISL_417100	02/29/2020	WA DOH / SFS	Chu et al
Spain/Canarias201939/2020	EPI_ISL_455326	02/29/2020	HUIGC / ISC	Iglesias-Caballero et al
USA/WA-S1488/2020	EPI_ISL_495663	03/08/2020	SFS / same	Nickerson et al
USA/WA-UW62/2020	EPI_ISL_415627	03/09/2020	UW Virology / same	Roychoudhury et al
USA/WA-UW-671/2020	EPI_ISL_476925	03/12/2020	UW Virology / same	Roychoudhury et al
USA/WA-UW197/2020	EPI_ISL_417346	03/12/2020	UW Virology / same	Roychoudhury et al
USA/WA-S540/2020	EPI_ISL_434300	03/24/2020	WA DOH / SFS	Chu et al
USA/WA-S579/2020	EPI_ISL_434339	03/24/2020	WA DOH / SFS	Chu et al
USA/WA-S390/2020	EPI_ISL_434150	03/27/2020	WA DOH / SFS	Chu et al
USA/WA-UW-2909/2020	EPI_ISL_486047	03/28/2020	UW Virology / same	Roychoudhury et al
USA/WA-UW-4243/2020	EPI_ISL_430939	04/01/2020	UW Virology / same	Roychoudhury et al
USA/WA-S333/2020	EPI_ISL_434093	04/04/2020	WA DOH / SFS	Chu et al
USA/WA-S712/2020	EPI_ISL_449882	04/07/2020	WA DOH / SFS	Chu et al
USA/WA-UW-5947/2020	EPI_ISL_430932	04/07/2020	UW Virology / same	Roychoudhury et al
USA/WA-S597/2020	EPI_ISL_438150	04/13/2020	SFS / same	Chu et al
USA/WA-S769/2020	EPI_ISL_449939	04/14/2020	WA DOH / SFS	Chu et al
USA/WA-S884/2020	EPI_ISL_463374	04/17/2020	WA DOH / SFS	Chu et al
USA/WA-S629/2020	EPI_ISL_438182	04/18/2020	WA DOH / SFS	Chu et al
USA/WA-S842/2020	EPI_ISL_463332	04/21/2020	WA DOH / SFS	Chu et al
USA/WA-S862/2020	EPI_ISL_463352	04/22/2020	WA DOH / SFS	Chu et al
USA/WA-S615/2020	EPI_ISL_438168	04/23/2020	SFS / same	Chu et al
USA/WA-S870/2020	EPI_ISL_463360	04/24/2020	WA DOH / SFS	Chu et al
USA/WA-S912/2020	EPI_ISL_463402	04/27/2020	WA DOH / SFS	Chu et al
USA/WA-S889/2020	EPI_ISL_463379	04/28/2020	WA DOH / SFS	Chu et al
USA/WA-S900/2020	EPI_ISL_463390	04/28/2020	WA DOH / SFS	Chu et al
USA/WA-S957/2020	EPI_ISL_463447	04/28/2020	WA DOH / SFS	Chu et al
USA/WA-S1007/2020	EPI_ISL_463497	04/30/2020	WA DOH / SFS	Chu et al
USA/WA-S1043/2020	EPI_ISL_463533	05/03/2020	WA DOH / SFS	Chu et al
USA/WA-S1159/2020	EPI_ISL_463649	05/04/2020	WA DOH / SFS	Chu et al
USA/WA-UW-8686/2020	EPI_ISL_501155	05/06/2020	UW Virology / same	Roychoudhury et al
USA/WA-S1310/2020	EPI_ISL_495760	05/14/2020	WA DOH / SFS	Nickerson et al

USA/WA-S1391/2020	EPI_ISL_495841	05/19/2020	WA DOH / SFS	Nickerson et al
USA/WA-S1413/2020	EPI_ISL_495863	05/20/2020	WA DOH / SFS	Nickerson et al
USA/WA-S1425/2020	EPI_ISL_495875	05/20/2020	WA DOH / SFS	Nickerson et al
USA/WA-S1562/2020	EPI_ISL_495988	05/26/2020	WA DOH / SFS	Nickerson et al
USA/WA-S1837/2020	EPI_ISL_496263	05/27/2020	WA DOH / SFS	Nickerson et al
USA/WA-S1851/2020	EPI_ISL_496277	05/27/2020	WA DOH / SFS	Nickerson et al
USA/WA-S1791/2020	EPI_ISL_496217	06/02/2020	WA DOH / SFS	Nickerson et al
USA/WA-S1940/2020	EPI_ISL_497368	06/02/2020	WA DOH / SFS	Nickerson et al
USA/WA-S1951/2020	EPI_ISL_497379	06/02/2020	WA DOH / SFS	Nickerson et al
USA/WA-S1995/2020	EPI_ISL_497423	06/04/2020	WA DOH / SFS	Nickerson et al
USA/WA-S2173/2020	EPI_ISL_497601	06/08/2020	WA DOH / SFS	Nickerson et al
USA/WA-S2298/2020	EPI_ISL_497726	06/10/2020	WA DOH / SFS	Nickerson et al
USA/WA-S2578/2020	EPI_ISL_497210	06/18/2020	WA DOH / SFS	Nickerson et al
USA/WA-UW-11263/2020	EPI_ISL_501094	06/18/2020	UW Virology / same	Roychoudhury et al
USA/WA-UW-13400/2020	EPI_ISL_501148	07/02/2020	UW Virology / same	Roychoudhury et al
USA/WA-UW-13306/2020	EPI_ISL_501135	07/03/2020	UW Virology / same	Roychoudhury et al
HKU-200823-001/2020	EPI_ISL_516798	03/26/2020	UHK / same	To et al
HKU-200823-002/2020	EPI_ISL_516799	08/17/2020	UHK / same	To et al
Nevada_Case_A	Pending	04/18/2020	Pending	Tillett et al
Nevada_Case_B	Pending	06/05/2020	Pending	Tillett et al
USA/WA-UW39/2020 (aka, InCoV139-March)	EPI_ISL_416456	03/06/2020	UW Virology / same	Roychoudhury et al
InCoV139-July	Pending	07/29/2020	ISB / same	Pending

Abbreviations: same = same originating and submitting laboratory; China CDC = National Institute for Communicable Disease Control and Prevention, Chinese Center for Disease Control and Prevention; WA DOH = Washington State Department of Health; SFS = Seattle Flu Study; UW Virology = University of Washington Virology Lab; PRMCE = Providence Regional Medical Center Everett; US CDC = Division of Viral Diseases, United States Centers for Disease Control and Prevention; HMC = Harborview Medical Center; SAViD = Area of Virology, Serology and Virology Division, New South Wales Health Pathology Randwick; CHCLB = Centre Hospitalier Compiagne Laboratoire de Biologie; NRCVRI = National Reference Center for Viruses of Respiratory Infections, Institut Pasteur, Paris; UHK = Department of Microbiology, The University of Hong Kong; HUIGC = Hospital Universitario Insular de Gran Canaria; ISC = Instituto de Salud Carlos III; ISB = Institute for Systems Biology.
